# Seroconversion to Pandemic (H1N1) 2009 Virus and Cross-Reactive Immunity to Other Swine Influenza Viruses

**DOI:** 10.3201/eid1710.110629

**Published:** 2011-10

**Authors:** Ranawaka A.P.M. Perera, Steven Riley, Siu K. Ma, Hua-Chen Zhu, Yi Guan, Joseph S.M. Peiris

**Affiliations:** The University of Hong Kong, Hong Kong Special Administrative Region, People’s Republic of China (R.A.P.M. Perera, S.K. Ma, H.-C. Zhu, Y. Guan, J.S.M. Peiris);; Imperial College, London, UK (S. Riley)

**Keywords:** human, immunity, swine, influenza, pandemic, H1N1, virus, dispatch

## Abstract

To assess herd immunity to swine influenza viruses, we determined antibodies in 28 paired serum samples from participants in a prospective serologic cohort study in Hong Kong who had seroconverted to pandemic (H1N1) 2009 virus. Results indicated that infection with pandemic (H1N1) 2009 broadens cross-reactive immunity to other recent subtype H1 swine viruses.

Pandemic (H1N1) 2009 was able to spread globally because it was antigenically divergent from contemporary human seasonal subtype H1N1 influenza viruses (*1*). Because we now recognize that pandemics can arise from influenza subtypes endemic in humans, it is essential that subtypes H1 and H3 swine viruses be considered potential future pandemic candidates, together with other avian virus subtypes such as H2, H5, or H9. Thus, it becomes imperative to investigate herd immunity in humans to swine and avian influenza viruses of subtypes H1 and H3.

Influenza virus subtypes H1 and H3 of diverse lineages are endemic in swine and are globally widespread. Eurasian avian-like swine H1 viruses are found in Europe; triple reassortant swine subtypes H1 and H3 viruses remain entrenched in North America (*2*). In China, we have demonstrated the co-circulation of these lineages together with classical swine (CS) subtype H1 viruses and also documented the emergence of antigenically variant reassortant viruses with gene segments of >2 of these lineages. We previously showed a lack of herd immunity in humans to some of these swine virus lineages in serum samples collected before the 2009 pandemic (*3*). However, the spread of pandemic (H1N1) 2009 worldwide may generate cross-reactive herd immunity to some of these swine virus lineages, making them less likely candidates for future pandemics. In this study, we assessed the relationship between seroconversion to pandemic (H1N1) 2009 and cross-reactive antibody responses to other major subtype H1 swine viruses in humans.

## The Study

Twenty-eight paired serum samples from a prospective serologic cohort study in Hong Kong, in which participants seroconverted to the pandemic (H1N1) 2009 virus, were selected to represent persons of diverse ages (median 39.5 years, range 8–74 years). Details of the serologic cohort have been reported elsewhere (*4*). The first (prepandemic or baseline) serum sample from each person was collected during July–August 2009, and the second (postpandemic or convalescent-phase) serum sample was collected during November 2009–February 2010. The peak of the first pandemic wave in Hong Kong occurred in September 2009. Subtype H1 swine influenza viruses, representative of CS, Eurasian avian-like swine (EA), triple reassortant swine (TRIG), pandemic (H1N1) 2009 viruses, and selected reassortants between these lineages with diverse antigenic profiles, were selected from our surveillance of swine influenza viruses in China (*3*). Relevant viruses from other geographic regions were also included. Each pair of baseline and convalescent-phase serum samples was tested for antibodies by microneutralization tests using each swine influenza virus. The profile of serologic responses to these swine viruses is shown in the Figure.

**Figure:**
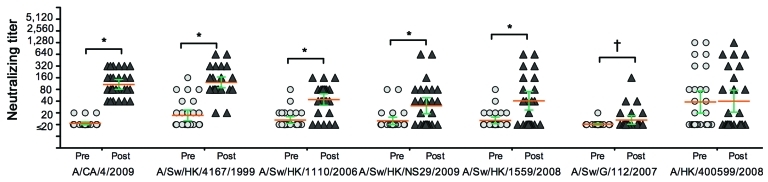
Neutralizing antibody titers to subtype H1 swine influenza viruses of the classical swine, North American triple reassortant, and Eurasian avian-like swine lineages in baseline (prepandemic [pre]) and convalescent-phase (postpandemic [post]) serum samples from 28 persons who seroconverted to pandemic (H1N1) 2009 infection, Hong Kong. Complete details on the serologic study cohort from which this subset is drawn are from (*4*). The pandemic A/California/4/2009 (H1N1) and seasonal influenza A/HK/400599/2008 (H1N1) viruses were used as controls. Orange lines indicate geometric mean titer; green error bars indicate 95% confidence intervals. *p<0.05; †p<0.01.

In accordance with our selection criteria, all 28 persons seroconverted (rise in antibody titer from <20 to >40) to the pandemic (H1N1) 2009 virus; follow-up antibody titers ranged from 40 to 320 (Figure). As expected, no serologic response occurred to seasonal influenza (H1N1) virus A/HK/400599/2008. Because the postpandemic serum samples were collected 2–5 months after the peak of the pandemic, waning of antibody titers over a few months is expected to be relatively modest (*5*). Notably, although few of the prepandemic serum samples tested had evidence of antibody titers >40 to any of the swine H1 viruses, the convalescent-phase serum sample of most persons had detectable antibody titers to other influenza viruses: CS (H1N1) Sw4167 (93% seropositive at a titer of >40; geometric mean titer [GMT] 121.9) and Sw1304 (86%; GMT 107.7); TRIG Sw1110 (75%; GMT 50); EA SwNS29 (46%; GMT 40), and an antigenically variant EA virus from Hong Kong that had acquired a nonstructural gene segment from TRIG virus by reassortment (57%; GMT 53) (Table).

**Table:** Seroprevalence and GMT for swine influenza viruses of H1 subtype in prepandemic and postpandemic serum specimens from 28 persons who seroconverted for pandemic (H1N1) 2009, Hong Kong*

Virus	Virus abbreviation	Virus lineage (abbreviation)	No. (%) seroconverters†			GMT	
			Prepandemic	Postpandemic		Prepandemic	Postpandemic
A/California/4/2009 (H1N1)	Cal4	Pandemic (Pdm)	0	28 (100)		10.77	107.7
A/Swine/HK/4167/1999 (H1N1)	Sw4167	Classical swine (CS)	6 (21)	26 (93)		17.24	121.9
A/Swine/HK/1304/2003 (H1N?)	Sw1304	Classical swine reassortant (CSr)	7 (25)	24 (86)		16.41	105
A/Swine/HK/1110/2006 (H1N2)	Sw1110	Triple reassortant (TRIG)	2 (7)	21 (75)		13.13	44.16
A/Swine/HK/NS29/2009 (H1N1)	SwNS29	Eurasian avian-like (EA)	2 (7)	13 (46)		12.50	30.46
A/Swine/HK/1559/2008 (H1N1)	Sw1559	Eurasian avian-like reassortant (EAr)	2 (7)	16 (57)		12.81	41.00
A/Swine/G/112/2007 (H1N1)	Sw112	Eurasian avian-like (EA)	0 (0)	3 (11)		10.25	13.13
A/Swine/HK/201/2010 (H1N1)	Sw201	TRIG reassortant (TRIGr)	3 (11)	21 (75)		12.2	48.76
A/HK/400599/2008 (H1N1)	400599	Seasonal influenza	12 (43)	12 (43)		38.07	40.00

*GMT, geometric mean titer. †Seroconverters were persons with antibody titer >40.

Because Hong Kong is a heavily urbanized environment, exposure of the study volunteers to live pigs during the 7-month study period is unlikely. Thus, the seroconversion to swine influenza viruses observed is not likely to be caused by infection with other swine influenza viruses. Notably, only 11% of these persons had neutralizing antibody titers to the EA virus SwG112, isolated in Ghent, Belgium. The difference between the EA viruses in Asia and Europe in this regard is worthy of further study. Because the hemagglutinin of EA virus is of avian origin, it is expected to cross-react poorly with subtype H1 of human or CS (derived from the 1918 pandemic H1 virus) derivation. What was unexpected was the observation that EA viruses isolated in China appear to manifest greater serologic cross-reactivity with pandemic (H1N1) 2009. Notably, little cross-reactivity occurred to 2 avian subtype H1 viruses isolated from wild birds in Hong Kong (data not shown).

A Poisson regression model of age as an indication of exposure for titer was used to look for evidence of age effects in both baseline and follow-up serum samples. The raw titers *t* (from the scale [<20, 20, 40, 80, …]) were transformed to outcome variable *x* (from the scale [0, 1, 2, 3, …]) in the following way: first, values of <20 were assigned the value of 10. Second, titers were divided by 10 and the logarithm taken (base 2). We used an uncorrected 95% statistical significance to test for preliminary evidence of an age effect. In the prepandemic serum samples, increasing age was significantly associated with increased antibody titers for pandemic (H1N1) 2009 (0.087; 95% confidence interval [CI] 0.002–0.720) and for TRIG virus 1110 (0.036; 95% CI 0.0009–0.062). Conversely, a significant negative relation with age for seasonal subtype H1N1 virus was found (−0.039; 95% CI −0.057 to −0.022). No significant age effects were found for other viruses. This age effect was lost in postpandemic infection serum samples, with the exception of antibody titers to the seasonal subtype H1N1 virus, which still had a negative association with age.

## Conclusions

In this study, we focused on defining the effects of seroconversion to pandemic (H1N1) 2009 on serologic cross-reactivity to other swine subtype H1 viruses. The next step should be to ascertain herd immunity to these swine influenza viruses in different population groups. We chose not to do this at this stage because the pandemic virus is still circulating among human populations, and seroprevalence is likely to continue to increase in different age groups over the next few years. Therefore, studying the effect of seroconversion to pandemic (H1N1) 2009 on cross-reaction to other swine influenza viruses would provide more meaningful information at this stage.

The results of our study suggest that the spread of pandemic (H1N1) 2009 in the population is broadening the serologic cross-reactivity and immunity in humans to other swine influenza viruses. However, gaps in immunity to selected swine influenza subtype H1 viruses remain (e.g., Sw112), at least as ascertained by neutralization antibody titers. We recognize, however, that neutralization tests do not capture all aspects of herd immunity in a population. Thus, our findings only serve to focus attention on the need for further study of population immunity to viruses such as Sw112. In general, these findings highlight the need for enhanced global surveillance of swine influenza viruses for the systematic assessment of human herd immunity to novel swine strains and to facilitate the development of routine (evidence-based) procedures for the ranking of known strains in terms of their pandemic risk.

## References

[1] Hancock K, Veguilla V, Lu X, Zhong W, Butler EN, Sun H, Cross-reactive antibody responses to the 2009 pandemic H1N1 influenza virus. N Engl J Med. 2009;361:1945–52. 10.1056/NEJMoa0906453 19745214

[2] Brockwell-Staats C, Webster RG, Webby RJ. Diversity of influenza viruses in swine and the emergence of a novel human pandemic influenza A (H1N1). Influenza Other Respi Viruses. 2009;3:207–13. 10.1111/j.1750-2659.2009.00096.x 19768134 PMC2746644

[3] Vijaykrishna D, Smith GDJ, Pybus OG, Zhu H, Bhatt S, Poon LLM, Long-term evolution and transmission dynamics of swine influenza A virus. Nature. 2011;473:519–22. 10.1038/nature10004 21614079

[4] Riley S, Kwok KO, Wu KM, Ning DY, Cowling BJ, Wu JT, Epidemiological characteristics of 2009 (H1N1) pandemic influenza based on paired sera from a longitudinal community cohort study. PLoS Med. 2011;8:e1000442 . 10.1371/journal.pmed.1000442 21713000 PMC3119689

[5] Hung IF, To KK, Lee CK, Lin CK, Chan JF, Tse H, Effect of clinical and virological parameters on the level of neutralizing antibody against pandemic influenza A virus H1N1 2009. Clin Infect Dis. 2010;51:274–9. 10.1086/653940 20575664

